# Biochemical Characterization and Effects of Cooking Methods on Main Phytochemicals of Red and Purple Potato Tubers, a Natural Functional Food

**DOI:** 10.3390/foods11030384

**Published:** 2022-01-28

**Authors:** Vincenzo D’Amelia, Giorgia Sarais, Giacomo Fais, Debora Dessì, Vittoria Giannini, Raffaele Garramone, Domenico Carputo, Sara Melito

**Affiliations:** 1 National Research Council of Italy, Institute of Biosciences and Bioresources (CNR-IBBR), Via Università 133, 80055 Portici, Italy; vincenzo.damelia@ibbr.cnr.it; 2 Food Toxicology Unit, Department of Life and Environmental Science, Campus of Monserrato, University of Cagliari, 09042 Cagliari, Italy; giacomo.fais@unica.it (G.F.); deboradessi95@gmail.com (D.D.); 3 Department of Agricultural Sciences, University of Sassari, Via Enrico de Nicola, 07100 Sassari, Italy; vgiannini@uniss.it (V.G.); smelito@uniss.it (S.M.); 4 Department of Agricultural Sciences, University of Naples, Via Università, 100, 80055 Portici, Italy; rgarramo@unina.it (R.G.); carputo@unina.it (D.C.)

**Keywords:** beneficial phytochemicals, antioxidants, agronomic performance, glycoalkaloids, potato, cooking methods

## Abstract

Potato is a staple food crop and an important source of dietary energy. Its tubers contain several essential amino acids, vitamins, minerals and phytochemicals that contribute to the nutritional value of this important product. Recently, scientific interest has focused on purple and red potatoes that, due to the presence of anthocyanins, may be considered as natural powerful functional food. The aim of this study was to evaluate the characteristics of pigmented varieties, the types of anthocyanins accumulated and the level of both beneficial phytochemicals (vitamin C and chlorogenic acids, CGAs) and anti-nutritional compounds (glycoalkaloids) following various cooking methods. The analyses described the presence of a mix of several acylated anthocyanins in pigmented tubers along with high level of CGA. The amount of antioxidants was differently affected by heat treatments according to the type of molecule and the cooking methods used. In some cases, the beneficial compounds were made more available by heat treatments for the analytical detection as compared to raw materials. Data reported here describe both the agronomic properties of these pigmented varieties and the effects of food processing methods on bioactive molecules contained in this natural functional food. They may provide useful information for breeders aiming to develop new varieties that could include desirable agronomical and industrial processing traits.

## 1. Introduction

Consumers are aware that a balanced diet is an important prerequisite for preventing chronic diseases and maintaining well-being. Over the years, there has been an increasing demand for functional foods able to supply several nutrients needed for a healthy diet [1]. Plant-based products are one of the fastest-growing market sectors of the food industry. Fruits and vegetables contain dietary fibers, large amounts of proteins, bioactive molecules, sterols and polyunsaturated fatty acids that positively affect target functions in the body [2]. Among cultivated plants, the potato (*Solanum tuberosum*) is a staple food crop worldwide. Its tubers are not only a source of carbohydrates but also contain essential amino acids, vitamins, minerals and important antioxidants [3,4]. Vitamin C (ascorbic acid) is among the most pronounced beneficial vitamins that contribute to the nutrient potential of potato tubers [5]. Vitamin C is well-known by consumers for its contribution to immune defense, but it is also a strong antioxidant and a co-factor of several regulatory enzymes [6]. Other phytonutrients, such as carotenoids, flavonoids and phenolic acids, increase the beneficial properties of potato-derived products thanks to their well-characterized antioxidant and human health-promoting properties [7]. Polyphenols are considered potent inhibitors of inflammations, obesity and cardiovascular disorders. Among polyphenols, chlorogenic acids (3-, 4- and 5-O-caffeoylquinic acids, CGAs) are widely accumulated in potato tubers and, along with flavonoids, represent a large part of the antioxidant repertoire of potatoes [4]. Anthocyanins are additional health-promoting compounds that can be found in potato varieties with purple and red tuber skin/flesh. They have important antioxidant properties, promote gut microbiota and reduce the glycemic index of starch [8,9]. We have recently provided evidence that diverse anthocyanin-rich extracts have different antiproliferative effects on hematological cancer cell lines [10]. This variability probably is caused by the different types of anthocyanins (together with other polyphenols) accumulated in tubers. Post-biosynthetic modifications such as methylation, acylation and polyglycosilation extend the types of anthocyanins produced and may affect the bioactive properties of these molecules [11]. Among Solanaceae, the potato can produce the widest range of anthocyanins with very high acylation levels. In fact, red potatoes contain an acylated form of pelargonidin, while purple or blue potatoes mainly contain an acylated form of petunidin, malvidin and peonidin [12]. Acylation with organic acids (commonly caffeic, p-coumaric and ferulic acids) generally enhances the physical chemical stability of anthocyanins during food technological processes, allowing to maintain the bioactive beneficial properties of these pigments in cooked foods [13].

The phytochemical composition of potato tubers is influenced by several factors. The genetic background plays a key role, with each potato variety possessing its nutritional properties. However, environmental–agronomic factors and their interaction with the genotype also impact the level and the type of health-promoting compounds naturally accumulated in the tubers [14]. Since potatoes are generally cooked before consumption, it is important to define the stability of the tuber phytochemicals during food processing, a novel trait to consider in breeding programs. In this regard, recently, Zhao et al. [15] highlighted the necessity to increase knowledge regarding how cooking methods can alter the phytochemical profiles of foods.

Bearing the above considerations in mind, we evaluated purple and red-fleshed potato varieties in terms of agronomical performances, major antioxidant molecules content (CGA, anthocyanins and vitamin C) and amount of anti-nutritional molecules (glycoalkaloids α-solanine and α-chaconine). In our study, both commercial and local varieties were used, and we examined how typical cooking methods could affect the content of the main phytochemicals.

## 2. Materials and Methods

### 2.1. Tuber Cultivation and Quality Evaluation

Three purple (Scano di Montiferro, Blue Star and Violet Queen) and one red (Magenta Love) potato variety was used in this study. Musica, a yellow-fleshed variety, was used as control. Principal characteristics of Musica, Scano di Montiferro, Violet Queen and Magenta Love are summarized in Table S1. Field trials were conducted during spring/summer (March to June) 2019 in Sardinia (Italy). The investigation was conducted in the experimental station of the University of Sassari in Ottava (40° N, 8° E, 81 m asl). Ottava is characterized by a well-drained soil with a clay-loam texture, a high content (>40%) of limestone and water retention capacity of 30%.

The climate of the experimental station is Mediterranean [16], characterized by fall–winter rains (535 mm) and dry summer. The annual mean temperature trends are in the range of 10 °C in January and 23.1 °C in August. During the experimental trial, the average minimum temperature in March (the coldest month) was 7.7 °C, while the warmest average temperatures were recorded in June (29 °C); total rain was 98 mm for the considered period. The experiment was set up in three replications with randomized blocks. Ten tubers per variety were planted in a single row with a spacing of 30 cm between tubers in the row and 70 cm between rows. During the growing season, plants were grown under irrigation and a fertilizer (N:P:K 18:22:16, 800 kg/ha) was given during soil preparation. Weeds were chemically controlled before potato plant emergence and by hand during the cropping season.

Tubers were harvested 120 days after planting when materials had already started senescence. Immediately after harvest, tubers from each variety were enclosed in paper bags and stored at 7 °C and 85–90% relative humidity for further evaluation. At harvest, tubers over 25 mm of diameter were collected and considered as marketable. Total tuber numbers for plant (TN), tuber yield (TY) and discarded (TD) was estimated. Tubers specific gravity (TSG) was estimated using 1 kg of tuber sample as the ratio weight in air/weight in water [17]. Chipping ability was also evaluated as a qualitative tuber parameter. A chipping test was performed by frying 10 longitudinally cut tuber slices from the center of each tuber. Two tubers for each variety were fried. Chip color was evaluated at harvest. To optimize the chipping test, 3 slices were washed in water before frying in soybean oil (180 °C). Tubers were considered completely fried when the oil stopped bubbling (1–3 min, depending on tuber specific gravity). A colorimetric scale, from 1 (very light) to 10 (very dark) was used to determine chipping ability [18]. Based on Carputo et al. [18], varieties with a score ≤4.5 were considered suitable for chipping.

### 2.2. Cooking Treatments

About 1 kg of tubers was selected based on similar weight and measures, cleaned and stored at room temperature until cooked, about two weeks after the harvest. Among the most common home preparation methods, four cooking methods (i.e., boiling, microwaving, baking, and frying) were tested to evaluate their effect on main polyphenols and antioxidant compounds. As for boiling, potatoes were peeled and cooked in 800 mL of water for 15 min after the water had come to boil. The samples were named as boiled peeled. For microwaving, non-peeled tubers were cut into small, evenly sized cubes and cooked in a microwave oven for 10 min at 800 W. These samples were named as microwaved non-peeled cut. For baking, non-peeled tubers were cut into small, evenly sized cubes and placed directly in the hot air oven and baked for 45 min at 180 °C. These samples were named as baked non-peeled cut. For frying, peeled and evenly sized cubes diced potatoes were added to 2 L of peanut oil in a domestic deep fryer at 170 °C for 2 min. These samples were named as fried. Raw samples were indicated as fresh non-peeled and fresh peeled. In both cases, potatoes were cut lengthwise into slices 2 mm thick and immediately frozen by liquid N_2._ Fresh peeled potatoes were peeled with a kitchen peeler in order to obtain peel 1 mm thick. The fresh peel weight ranged from 10 to 15% of the whole weight. All samples were freeze-dried before chemical analysis.

### 2.3. Chemicals

Acetonitrile was of HPLC grade and was purchased from Sigma (Milano, Italy). Orthophosphoric acid (ACS ISO, for analysis, 85%) and ethanol (ACS-Reag. Ph.Eur.) were purchased from Carlo Erba Reagents S.r.l. (Milano, Italy). Water was distilled and filtered through a Milli-Q apparatus (Millipore, Milan, Italy). Standards of malvidin-3-O-glucoside, cyanidin, ascorbic acid, chlorogenic acid, α-solanine and α-chaconine were purchased from Sigma (Milano, Italy). C18 SPE columns (500 mg 3 mL, Supelco, Bellefonte, PA, USA) were used.

### 2.4. Determination of Total Anthocyanin Content

Total anthocyanin content (TAC) was determined using a spectrophotometric pH differential protocol according to Giusti and Wrolstad [19]. This method allows to determine total monomeric anthocyanin content on the basis of reversible structural changes in the anthocyanin chromophore between pH 1.0 and 4.5. The difference in the absorbance of the pigments at 520 nm is proportional to the pigment concentration. Briefly, fresh tubers were cut into small pieces and homogenized through a standard household blender for 3 min. The mixed sample was soaked with 70% ethanol and, after 12 h, the alcoholic extract was separated by filtration. Finally, the solution was divided into two equal aliquots and processed in triplicate. The first one was mixed with potassium chloride buffer in order to reach pH 1, and the second one was mixed with sodium acetate buffer to reach pH 4.5. The absorbance of these solutions was registered at 520 nm against a blank solvent. Because solutions were clarified by centrifuging at 4000 rpm before analysis, it was unnecessary to measure the absorbance at 700 nm and to subtract it from the absorbance at the maximum absorption wavelength to correct for haze. Total anthocyanin content, expressed as mg of cyanidin equivalent kg^−1^ of dry matter (DM), was calculated using the equation: TAC = [(A_1_ − A_2_) × MW_Cyan_ × DF]/W where A_1_ is the absorbance in potassium chloride buffer (pH 1), A_2_ is the absorbance in sodium acetate buffer (pH 4.5), MW is the molecular weight of cyanidin, DF is the dilution factor and W is sample weight.

### 2.5. Single Anthocyanins and Chlorogenic Acid Analyses

Single anthocyanins and chlorogenic acid extractions were carried out following the protocol developed by Sarais et al. [20]. According to this method, an aliquot of samples thinly pulverized was extracted with a 70% aqueous methanol solution. An ultrasonic extraction experiment was carried out using an ultrasonic system (Ultracleaner 040S, Be-Right (Medical) Co., Ltd., Foshan, China) for 60 min. In all experiments, the temperature in the beaker was maintained under 25 °C using a thermostatic controller to avoid polyphenol degradation. Samples were finally collected, centrifuged at 4000 rpm, and supernatants were utilized to determine the contents of anthocyanins and chlorogenic acid. An Agilent 1100 system consisting of a G1311A quaternary pump, a G1313A rheodyne injector, a G1316A thermostated column compartment, a G1322A degasser, and coupled with a DAD detector UV 6000 (Thermo Finnigan, Milan, Italy) was employed to develop the chromatographic method. Analyses were performed using a Kinetex column (5u, C18, 100 A; Phenomenex, Torrance, CA, USA), eluted with mobile phases A (acetonitrile) and B (H_2_O with 0.22 M phosphoric acid). A linear gradient program at a flow rate of 0.4 mL/min was used: 0–30 min from 5 to 10% (A); 30–35 min from 10 to 15% (A); 35–70 min from 15 to 30% (A), 70–100 min from 30 to 90% (A), then to 100% (A) up to 120 min. A post-time of 20 min was used to allow the column to equilibrate before the next sample injection. Detection was carried out at 280 nm for chlorogenic acid and 520 nm for anthocyanins. Extracts were directly injected onto an HPLC after dilution with phosphoric acid 0.22 M. Concentration of each active ingredient quantified, calculated by comparing the area of the sample with those of the reference standard, was expressed as mg kg^−1^ of dry weight. All tests were carried out in triplicates and the standard deviation was calculated for all data.

### 2.6. Ascorbic Acid Content (Vitamin C)

A 1 g portion of potatoes thinly pulverized was taken for each preparation technique and was extracted twice with water (10 mL) in an ultrasonic bath for 15 min (Ultracleaner). The pooled extracts were evaporated to dryness under vacuum, and the residues were dissolved in aqueous solution phosphoric acid 0.22 M. The solution was filtered through a 0.45 μm nylon membrane (Millipore) prior to injection into the HPLC-DAD. Separation for qualitative and quantitative analysis of the ascorbic acid was performed according to the method described for anthocyanins and chlorogenic acid [20]. Detection was carried out at 254 nm according to maximum absorbance wavelength. Results were expressed as mg kg^−1^ of dry weight. All tests were carried out in triplicates and the standard deviation was calculated for all data.

### 2.7. Chaconine and Solanine Content

Modified methods of Bodart et al. [21] were used to extract α-solanine and α-chaconine. Extraction of glycoalkaloids was performed with three portions of boiling methanol–chloroform (95: 5, *v*/*v*) for 15 min under continuous agitation. The solution was quantitatively transferred to a C18 SPE column activated and conditioned with 3 mL of methanol. The column was filled up with the sample and α-solanine and α-chaconine were eluted with 3 mL of aqueous solution of acetonitrile 70%. The eluate was directly injected into the HPLC–DAD. The HPLC system, solvents and column were the same for anthocyanins analysis. The separation of the two glycoalkaloids was achieved with the following gradient program with a flow rate of 0.4 mL/min: 0–10 min from 25% to 30% (A); 10–20 min from 30% to 35% (A). A post-time of 5 min was set up. Detection was carried out at 205 nm and results were expressed as mg kg^−1^ of dry weight for both α-solanine and α-chaconine. All tests were carried out in triplicates and the standard deviation was calculated for all data.

### 2.8. Stock Standard Solution of the Analytes and Calibration Curve

Stock solutions of individual standards were prepared by dissolving 10 mg of each compound in 10 mL of methanol to a final concentration of 1000 mg L^−1^. Two different calibration curves were created: the first one was employed to quantify anthocyanins, chlorogenic acid and ascorbic acid, the second one for glycoalkaloids quantification. For this reason, working standard solutions were prepared by making a series of varying dilutions of the different stock standard solutions. Known volumes of each stock standard were transferred into the same volumetric flasks and diluted by adding 0.22 M aqueous solution phosphoric acid to obtain mixed reference solutions in the range of 0.02–20 mg L^−1^. All standard solutions were stored in the dark at −20 °C until usage. A quantitative determination using a multiple-point external standard method was performed at each wavelength of the maximum UV–Vis absorbance for all analytes. A five-point calibration curve of a standard mix of anthocyanins, chlorogenic acid and ascorbic acid was prepared in the starting mobile phase. The same protocol was employed for α-chaconine and α-solanine calibration curve preparation. Curves were created by plotting the peak area versus the nominal concentration of the analytes. Due to lack of standards, anthocyanin concentrations were calculated using malvidin 3-O-glucoside as reference and were expressed as its equivalent.

### 2.9. Statistical Analysis

One-way analysis of variance (ANOVA) was performed using JMP 7 software (SAS Institute, Cary, NC, USA), to evaluate TY, TSG and CC. When a significant F was found (*p* < 0.05), separation of means was accomplished by Tukey’s post hoc multiple comparison test. For each trait, single degree of freedom contrasts were used to compare the mean values among varieties to the mean of varieties and to varieties individually.

## 3. Results and Discussion

Consumers have become increasingly careful about healthy diets, and foods enriched in well-known beneficial phytochemicals are nowadays particularly appreciated. This is leading to an increase in demand of red/blue potatoes, rich sources of vitamin C, minerals and phenolic acids. They also contain anthocyanins with marked health-promoting properties [22,23]. This re-discovered functional food was examined in this study. We critically explored the phytochemical compositions of colored tubers and how these phytochemicals are affected by traditional cooking methods. A preliminary agronomic evolution of pigmented tuber varieties was carried out. Differences in yield (TY), tuber specificity gravity (TSG) and chipping ability (expressed as chipping category color, CC), were found among our varieties [24,25]. Overall, as previously reported by other authors [26], pigmented varieties showed a lower yield compared to Musica (Table 1). Though no significant differences were measured in terms of TSG, we found that all pigmented flesh varieties showed higher dry matter content than Musica, confirming what was previously observed [27]. All the commercial varieties showed a CC value lower than 4.5. Only local landrace Scano di Montiferro gave unacceptably dark chips, as indicated by the CC value of 7.00.

Planting colored flesh potatoes can provide an opportunity to diversify potato production and to increase the potential of potato tubers as “functional food”. In the second stage of our work, tubers of the above varieties were biochemically analyzed before and after cooking, and results are reported in the following paragraphs. For the metabolic analyses, we also added the variety Blue Star to aid comparisons with previous studies. In fact, Blue Star has been already well characterized for chemical composition and potential industrial uses [10,28].

### 3.1. Quantitative Analysis of Chlorogenic Acid in Raw Colored and Yellow Tubers and the Effect of Domestic Cooking Methods

**Figure 1:**
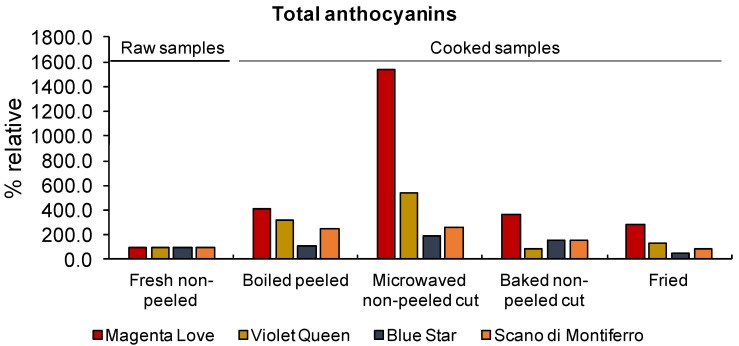
The effects of different cooking methods on total anthocyanin content in purple and red tubers expressed as percentage with respect to fresh non-peeled samples (raw samples).

Phenolic compounds largely contribute to the antioxidant weaponry of potato tubers [29]. The most abundant phenolic compounds in our samples belonged to the phenolic acids class and they were all represented by isomers of monosubstituted esters of caffeic and quinic acid, named chlorogenic acids (CGAs). CGA content varied greatly among varieties studied here, ranging from 81.1 ± 4.3 to 3724.8 ± 152.5 mg kg^−1^_DM_ (Table 2). Magenta Love showed the highest quantity of CGAs (3724.8 ± 152.5 mg kg^−1^_DM_) followed by Violet Queen (1966.5 ± 74.1 mg kg^−1^_DM_), Scano di Montiferro (1685.6 ± 57.9 mg kg^−1^_DM_) and Musica (81.1 ± 4.3 mg kg^−1^_DM_). The lower amount of CGA detected in the yellow-fleshed Musica can be explained by the shared molecular regulation between anthocyanins and CGA, which is promoted by the same R2R3-MYB transcription factor [30]. In other words, the presence of anthocyanins in potato tubers may mark also an increment of additional beneficial phenolic compounds, including CGAs, due to the upregulated activity of the same transcriptional regulator. According to de Andrade Lima et al. [31], tuber peel largely contributes to total phenolic compounds. In our samples, peels contributed 30% to the total amount of CGA detected, especially in the tubers of colored varieties (i.e., Magenta Love, Scano di Montiferro and Violet Queen). In Musica, no changes in total amount of CGA between peeled and unpeeled tubers were found, suggesting that CGA accumulation was limited to the flesh (Figure 1, Table S2). Purple/red peels can be considered a valuable by-product as a rich source of anthocyanins and CGA [32]. Agricultural and industrial wastes enriched in anthocyanins and polyphenols have already shown a great potential to be re-used as a source of antioxidants in the idea of circular economy. For example, eggplants, purple oranges and grape pomace have been used as sources of anthocyanins for different purposes [33,34,35]. Potato peels represent 15–40% of by-products after potato processing [36]. Thus, it may represent a valuable material that can be used for anthocyanins extraction. Peels from potato may be even directly used as plastic films for packaging [37] and for the production of the so-called smart films (i.e., with bioactive film monitoring food quality and extending shelf life of products) [38].

As regards the effect of food processing, the pattern of CGA content among varieties remained generally conserved after and before cooking (Table 2). Magenta Love showed the highest amount of CGA, followed by Scano di Montiferro, Violet Queen, Blue Star and Musica in both raw and cooked samples. We registered an overall increase in CGA concentration following microwaving in all varieties. This was particularly evident in Magenta Love, whose CGA content increased from about 4000 to 14,000 mg kg^−1^ (Table 2). Degradation in CGA content after frying was observed in Violet Queen and Scano di Montiferro, with a reduction of 30% and 20%, respectively. Frying usually has a negative impact on total phenolic content, and in particular on CGA. Furrer et al. [39] reported an average reduction of 35% of different CGA fractions (3-CQA, 4-CQA and 5-CQA) following frying.

### 3.2. Anthocyanin Fraction Variability and the Effect of Domestic Cooking Methods

Anthocyanins are responsible for the red and purple color of potatoes. Cyanidin and peonidin contribute more to red hues, pelargonidin has more orange color, while blue-violet colors are typical of delphinidin, petunidin and malvidin [40]. As reported in Table 3, both red and purple-fleshed potato varieties contained high levels of total anthocyanins, ranging from 1688.3 ± 70.2 to 131.3 ± 10.7 mg kg^−1^_DM_ in Violet Queen and Blue Star, respectively. In most cases, peeled samples showed a higher level of anthocyanins than non-peeled ones. Though it is not easy to give a clear explanation of these results, similar data were observed by Lachman et al. [41] with different potato varieties.

Colored potato varieties, compared to other Solanaceous crops, display all the six of the most common anthocyanidins [42]. The anthocyanin profile of our raw and peeled samples is reported in Table 4 (for Violet Queen, Scano di Montiferro and Blue Star) and in Table 5 (for Magenta Love).

The types of anthocyanins detected did not differ between flesh and the entire tubers, suggesting a shared control of the enzymatic repertoire between different tissues of the same variety. Violet Queen extracts were characterized by high amounts of petunidin derivatives, especially petunidin 3-O-p-coumaroyl rutinoside 5-O-glucoside (360.9 ± 13.0 mg kg^−1^_DM_). Anthocyanin composition of tubers of Scano di Montiferro was mainly ascribable to malvidin 3-O-p-coumaroyl-rutinoside-5-O-glucoside (350.1 ± 15.5 mg kg^−1^_DM_), whereas small amounts of petunidin derivatives were detected (Table 4). In Blue Star, we found a more equilibrated balance between petunidin and malvidin derivatives. Petunidin and malvidin differ for a single or double O-methylation at 3′-OH or both 3′ and 5′- OH on a delphinidin precursors (Figure S1). It seems that the same methyltransferase is involved in the methylations of both positions, as suggested in grapevine [43]. Differences observed in the content of the main anthocyanidin molecules, petunidin and malvidin, may be due to a different enzymatic efficiency of methyltransferases across the different varieties. In other words, the different varieties can hold different methyltransferase isoforms that can exert different abilities in methylating delphinidin on the two different positions. This interesting aspect could be better clarified through genetic and molecular studies. The anthocyanins profile of Magenta Love was different, characterized by diverse types of anthocyanin molecules, not comparable with that of the other varieties. Indeed, it was characterized mainly by the presence of pelargonidin derivatives, four times more concentrated than peonidin (Table 5), with pelargonidin 3-O-p-coumaroyl-rutinoside-5-O-glucoside being the most abundant anthocyanin (129.1 ± 9.0 mg kg^−1^_DM_). The presence of typical red pigments in tubers of Magenta Love is probably due to the lack of functionality of the locus P (p) which encodes for flavonoid 3′,5′-hydroxylase [44]. Varieties with the specific allelic combination (i.e., *pppp*) may have a red-directed metabolic flux towards red (pelargonidin and peonidin) rather than purple anthocyanins. The anthocyanidin profile of Magenta Love and Blue Star is in accordance with previous data [29]. However, we detected a different glycosylation and acylation of the anthocyanidins (e.g., preferences for acylation with different hydroxycinnamic acids), corroborating the hypothesis that anthocyanin decorations can be influenced by the environment [45].

The evaluation of total anthocyanin content following different cooking treatments is reported in Figure 1. We expressed the total content of anthocyanins as relative percentage compared to raw potatoes (fresh non-peeled, 100% reference). Absolute values for each type of anthocyanin are reported in Tables S2 and S3. We found that, generally, cooking treatments positively affected the amount of anthocyanins (Figure 1). Frying was the only cooking method that induced a loss of total anthocyanin (about 46%) in Blue Star. Microwaving led to an increase in total anthocyanins content in all varieties tested, with a maximum value in Magenta Love, that showed a concentration 15-fold higher than that of raw samples (fresh non-peeled potatoes). Magenta Love also displayed the highest increase in total anthocyanins after all cooking treatments, followed by Violet Queen and Scano di Montiferro. For all varieties, baking and boiling treatments resulted in a slightly increment of pigment concentration. These results, along with previous studies [46,47], further suggest that anthocyanins may not be readily degraded during thermal processing. Our results are also in accordance with those of Lachman [46], who reported that heating treatments increase the concentration of anthocyanins measured in colored potato tubers. As observed by Lemos et al. [47], this could be explained with a modification of the tuber flesh texture correlated to the disruption of the cells walls during cooking treatments. It would allow a better and easier extraction of anthocyanins and phenolic compounds from vegetables.

### 3.3. Quantitative Analysis of Vitamin C in Colored and Yellow Tubers and the Effect of Domestic Cooking Methods

**Figure 2:**
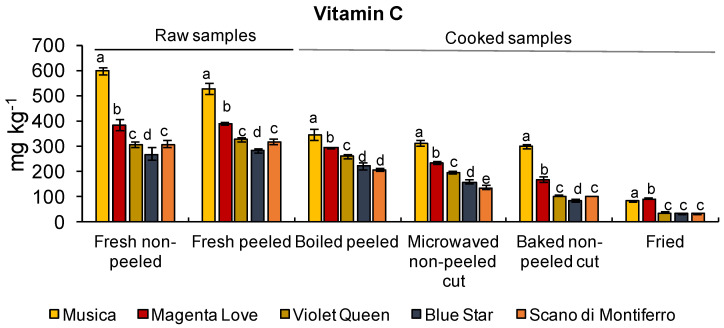
The content of vitamin C (mg kg^−1^) in raw peeled and non-peeled potato tubers and after different cooking methods (x-axis). Values are means ± SD (*n* = 3). Means denoted by the same letter, within the same group described on the x axis, did not differ significantly at *p* ≤ 0.05 according to Tukey’s multiple range test.

In order to check the nutritional value of the tubers in terms of other phytochemicals, we monitored the level of vitamin C and the amount of α-chaconine and α-solanine, two potato anti-nutritional glycoalkaloids. Recommended intakes of vitamin C in EU vary between 95 and 110 mg per day [48]. All varieties analyzed here contained a good concentration of vitamin C, with Musica showing the highest level (597.8 ± 14.1 mg kg^−1^_DM_; Figure 2, Table S4). Among pigmented varieties, the content of vitamin C content was similar, with values that ranged between 268.3 ± 26.7 and 382.9 ± 21.6 mg kg^−1^_DM_. In a larger sample size, also Külen et al. [49] observed that pigmented varieties had lower vitamin C content than unpigmented ones. This opens an interesting avenue for future research on the molecular regulatory network underlying the negative regulation between anthocyanin and vitamin C content. Based on our results, peels generally did not contribute to the total amount of vitamin C. This is in line with the results of Lachman et al. [41], who reported that peeling affected the content of this vitamin only to a small degree.

We observed that the concentration of vitamin C generally decreased after cooking (Figure 2). These results confirm that culinary treatments, such as boiling, microwaving, frying and baking cause loss of this vitamin [50]. The greatest loss was observed after frying, that reduced the total amount of vitamin C to a value below 100 mg kg^−1^. Scano di Montiferro was the worst variety in this sense. It showed 89% of vitamin C loss as compared to raw samples (fresh peeled). The boiling and microwaving cooking methods led to the lowest loss of vitamin C, ranging from 12.7% (Violet Queen) up to 45% (Musica) for boiling and from 34.0% (Magenta Love) up to 51.8% (Scano di Montiferro) for microwaving.

### 3.4. Quantitative Analysis of Glycoalkaloids in Colored and Yellow Tubers and the Effect of Domestic Cooking Methods

**Figure 3:**
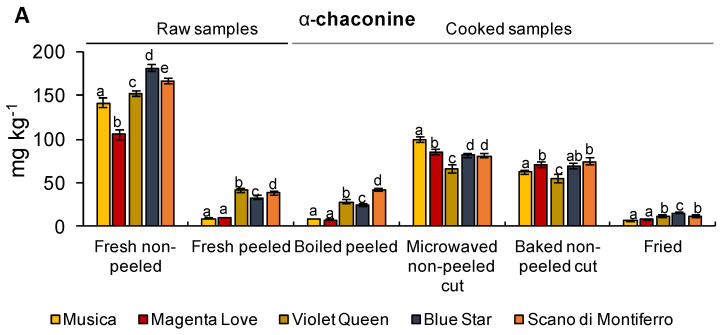

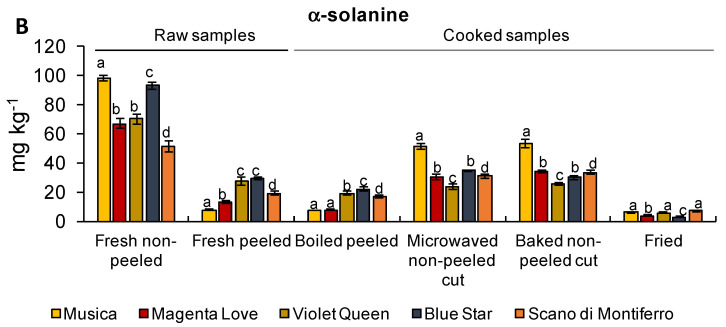
The content of glycoalkaloids (mg kg^−1^) in raw peeled and non-peeled potato tubers and after different cooking methods (x-axis). (**A**) α-chaconine; (**B**) α-solanine. Values are means ± SD (*n* = 3). Means denoted by the same letter, within the same group described on the x-axis, did not differ significantly at *p* ≤ 0.05 according to Tukey’s multiple range test.

Besides these beneficial compounds, tubers accumulate specialized metabolites with potential adverse effects on humans. For example, α-chaconine and α-solanine, representing about 95% of the glycoalkaloids in tubers of cultivated potatoes, are considered toxic in a dose dependent manner. For food safety purposes, different European and extra-European countries suggested that glycoalkaloid levels should be lower than 100–200 mg kg^−1^ of potato. At the EU community level, as reported in the EFSA scientific opinion, the maximum levels of glycoalkaloids in potatoes are still to be set [51]. Glycoalkaloid content is highly related to the genetic background of plant materials [52]. In our raw samples, α-chaconine was always more abundant than α-solanine but below the prescribed limit (Figure 3A,B, Tables S5 and S6). The analysis conducted by Musita et al. [53] suggested that the difference in the amount of the two glycoalkaloids is generally conserved among potato tubers, with α-chaconine always more abundant than α-solanine. On the contrary, the total amount of glycoalkaloids can highly vary between the different varieties [53]. Our results provided evidence that a large contribution to the amount of these alkaloids comes from the peels, confirming data from the literature [54]. In our work, α-chaconine and α-solanine content reduced after different cooking treatments (Figure 3A,B). A strong reduction was observed after frying and boiling. In particular, after boiling, we observed an average reduction of 80% and 65%, respectively, for α-solanine and α-chaconine, while frying reduced the average amount of both glycoalkaloids by about 90%. This is in line with what was observed by Nie et al. [55], who showed a reduction in glycoalkaloid content of about 94% in fried chips compared to raw tubers. However, some authors suggested that the reduction in glycoalkaloid content in chips is caused by the whole cooking process (i.e., blanching and drying) rather than only by frying. As also observed for the other compounds, microwave and baking were the least efficient to reduce α-solanine and α-chaconine. For example, microwave reduced by only 30% the content of α-chaconine in the tuber flesh of Musica (from 141 to 99 mg kg^−1^), while baking reduced the amount of α-solanine by only 35% in Scano di Montiferro (from 51 to 33 mg kg^−1^). These results are in accordance with EFSA, that reported that microwave and oven baking of unpeeled potatoes may cause a reduction in GA content by 3–45% and by 20–50%, respectively [51].

## 4. Conclusions

Besides being an incredible source of energy, potato tubers contain several phytochemicals that help to meet the recommended dietary allowances for vitamins and antioxidants. Purple and red potatoes can be considered, for several aspects, a natural functional food. Our results indicated that the presence of anthocyanins associates with a high level of chlorogenic acid, another phenolic antioxidant that contributes to increasing the nutritional value of potato tubers. However, our results also reported that there is a lower amount of vitamin C in pigmented potatoes and cooking even further reduced this concentration. Considering that also previous studies evaluated the negative association between anthocyanins and vitamin C, future studies are needed to understand the biochemical and molecular association between these two important biochemical pathways.

## Figures and Tables

**Table 1:** Results for the evaluation of different potato varieties.

Variety	TY	TSG	CC
Musica	0.59 ± 0.13 ^a^	1.069 ± 0	4.00 ± 0.58 ^b^
Magenta Love	0.14 ± 0.08 ^b^	1.078 ± 0	2.00 ± 0 ^c^
Scano di Montiferro	0.20 ± 0.01 ^b^	1.095 ± 0	7.00 ± 0.58 ^a^
Violet Queen	0.21 ± 0.04 ^b^	1.079 ± 0	4.00 ± 0 ^b^

^a–c^ Means denoted by the same letter did not differ significantly at *p* ≤ 0.05 according to Tukey’s multiple range test. Tuber yield (TY, kg of tubers per plant), tuber specific gravity (TSG) and chip category color (CC) of all tested genotypes. Chip category color was evaluated at harvest (direct). For each trait, the average value and the standard deviation was reported.

**Table 2:** Content of chlorogenic acid and isomers (CGAs) in raw and cooked potatoes expressed as mg kg^−1^ of DM.

Variety	Fresh Non-Peeled	Fresh Peeled	Boiled Peeled	Microwaved Non-Peeled Cut	Baked Non-Peeled Cut	Fried
Musica	81.1 ± 4.3 (a)	83.5 ± 12.2 (a)	15.2 ± 1.9 (a)	595.7 ± 16.4 (a)	121.7 ± 18.9 (a)	226.3 ± 21.2 (a)
Magenta Love	3724.8 ± 152.5 (b)	2615.6 ± 198.6 (b)	8074.8 ± 221.5 (b)	13,530.3 ± 315.6 (b)	3668.8 ± 88.7 (b)	2776.1 ± 51.8 (b)
Violet Queen	1966.5 ± 74.1 (c)	1382.7 ± 101.8 (c)	4315.5 ± 84.6 (c)	3748.7 ± 86.9 (c)	2457.7 ± 95.6 (c)	1002.3 ± 16.7 (c)
Blue star	387.5 ± 33.6 (d)	361.4 ± 24.1 (d)	388.7 ± 21.3 (d)	3115.7 ± 25.7 (d)	1175.6 ± 102.3 (d)	371.0 ± 3.9 (d)
Scano di Montiferro	1685.6 ± 57.9 (c)	1059.9 ± 31.2 (c)	2119.8 ± 14.2 (e)	5785.7 ± 66.4 (e)	1594.3 ± 109.7 (e)	865.0 ± 11.2 (c)

Values are means ± SD (*n* = 3). Statistical analyses were undertaken by comparing CGA content across varieties. Means denoted by the same letter did not differ significantly at *p* ≤ 0.05 according to Tukey’s multiple range test.

**Table 3:** Total anthocyanin content in raw potatoes expressed as mg kg^−1^ of DM (mean + SD; *n* = 3).

Variety	Raw Non-Peeled	Raw Peeled
Musica	nd	nd
Magenta Love	471.2 ± 30.1	1054.1 ± 1.4
Violet Queen	1688.3 ± 70.2	1311.0 ± 115.7
Blue Star	131.3 ± 10.7	136.7 ± 15.9
Scano di Montiferro	984.5 ± 37.5	1213.0 ± 50.1

nd = not detectable.

**Table 4:** Single anthocyanin content in raw purple potato tubers. Values are expressed as mg kg^−1^ of DM (mean + SD; *n* = 3).

Variety		Petunidin 3-O rutinoside *	Petunidin 3-O-caffeoyl-rutinoside-5-O-glucoside *	Petunidin 3-O-p-coumaroyl rutinoside 5-O-glucoside *	Petunidin 3-O-feruloyl rutinoside 5-O-glucoside *	Malvidin 3-O-p-coumaroyl-rutinoside-5-O-glucoside *	Malvidin 3-O-p-feruloyl-rutinoside-5-O-glucoside *
Violet Queen	Fresh non-peeled	19.4 ± 2.0 b	27.4 ± 1.3 b	360.9 ± 13.0 b	17.0 ± 0.8 c	98.7 ± 3.2 b	12.8 ± 0.9 b
	Fresh peeled	17.6 ± 1.0 b	60.5 ± 2.9 b	912.5 ± 33.9 b	31.7 ± 0.4 c	177.0 ± 3.8 b	22.3 ± 1.6 a
Blue Star	Fresh non-peeled	<0.5 a	11.3 ± 0.8 a	24.8 ± 1.3 a	11.4 ± 0.7 b	15.5 ± 0.7 a	11.5 ± 1.0 a
	Fresh peeled	<0.5 a	15.4 ± 0.8 a	32.9 ± 1.8 a	8.9 ± 0.3 b	11.5 ± 0.9 a	8.9 ± 0.6 b
Scano di Montiferro	Fresh non-peeled	31.3 ± 1.8 c	16.2 ± 1.0 a	13.3 ± 1.0 a	N.D. a	350.1 ± 15.5 c	29.5 ± 1.9 c
	Fresh peeled	36.7 ± 2.0 c	21.9 ± 1.1 a	42.3 ± 1.4 a	N.D. a	443.1 ± 17.3 c	34.8 ± 1.9 c

* Anthocyanins concentrations were expressed as malvidin 3-O-glucoside equivalent; a–c: Means denoted by the same letter did not differ significantly at *p* ≤ 0.05 according to Tukey’s multiple range test.

**Table 5:** Single anthocyanin content in raw red potato tubers. Values are expressed as mg kg^−1^ of DM (mean + SD; *n* = 3).

Variety		Petunidin 3-O-rutinoside-5-glucoside *	Pelargonidin 3-O-rutinoside *	Pelargonidin 3-O-p-caffeoyl- rutinoside-5-O-glucoside *	Pelargonidin 3-O-cis-p-coumaroyl-rutinoside-5-O-glucoside *	Pelargonidin 3-O-p-coumaroyl- rutinoside-5-O-glucoside *	Peonidin 3-O-p-coumaroyl- rutinoside-5-O-glucoside *	Pelargonidin 3-O-p-feruloyl- rutinoside-5-O-glucoside *	Peonidin 3-O-p-feruloyl- rutinoside-5-O-glucoside *
Magenta Love	Fresh non-peeled	<0.5 mg/kg	<0.5	23.8 ± 1.1	26.7 ± 2.4	129.1 ± 9.0	30.0 ± 2.0	25.3 ± 1.0	20.6 ± 1.0
	Fresh peeled	54.0 ± 4.1	19.4 ± 1.9	39.0 ± 2.5	82.3 ± 6.5	573.6 ± 21.8	90.2 ± 6.8	42.6 ± 2.9	16.2 ± 0.6

* Anthocyanins concentrations were expressed as malvidin 3-O-glucoside equivalent.

## Data Availability

The datasets generated for this study are available on request to the corresponding author. The study did not report any data.

## Supplementary Materials

The following are available online at www.mdpi.com/article/10.3390/foods11030384/s1, Table S1: Tuber characteristics of the potato genotype tested in the experimental field. For each genotype, the name, origin, tuber skin color (TSC), tuber flesh color (TFC), tuber shape (TS), eye and stolon characteristics are reported, Table S2: Anthocyanin profile in raw and cooked potatoes expressed as mg kg^−1^ of DM. Values are means ± SD (*n* = 3). Means denoted by the same letter, within the same group described on the x axis, did not differ significantly at *p* ≤ 0.05 according to Tukey’s multiple range test, Table S3: Anthocyanin profile in raw and cooked potatoes expressed as mg kg^−1^ of DM (mean ± SD; *n* = 3), Table S4: Content of vitamin C in raw and cooked potatoes expressed as mg kg^−1^ of DM (mean ± SD; *n* = 3), Table S5: Content of α-chaconine in raw and cooked potatoes expressed as mg kg^−1^ of DM (mean ± SD; *n* = 3), Table S6: Content of α-solanine in raw and cooked potatoes expressed as mg kg^−1^ of DM (mean ± SD; *n* = 3), Figure S1: General structure of anthocyanins accumulated in potato tubers.
