# Supplementary material for: Biochemical Characterization and Effects of Cooking Methods on Main Phytochemicals of Red and Purple Potato Tubers, a Natural Functional Food
Source: Foods. 2022 Jan 28;11(3):384. doi: 10.3390/foods11030384 (PMC8834363; doi:10.3390/foods11030384)
Supplement: Supplementary file 1 [file foods-11-00384-s001.zip › Supplementary figure_R1.pdf]

Figure S1. General structure of anthocyanins in potato samples tested.

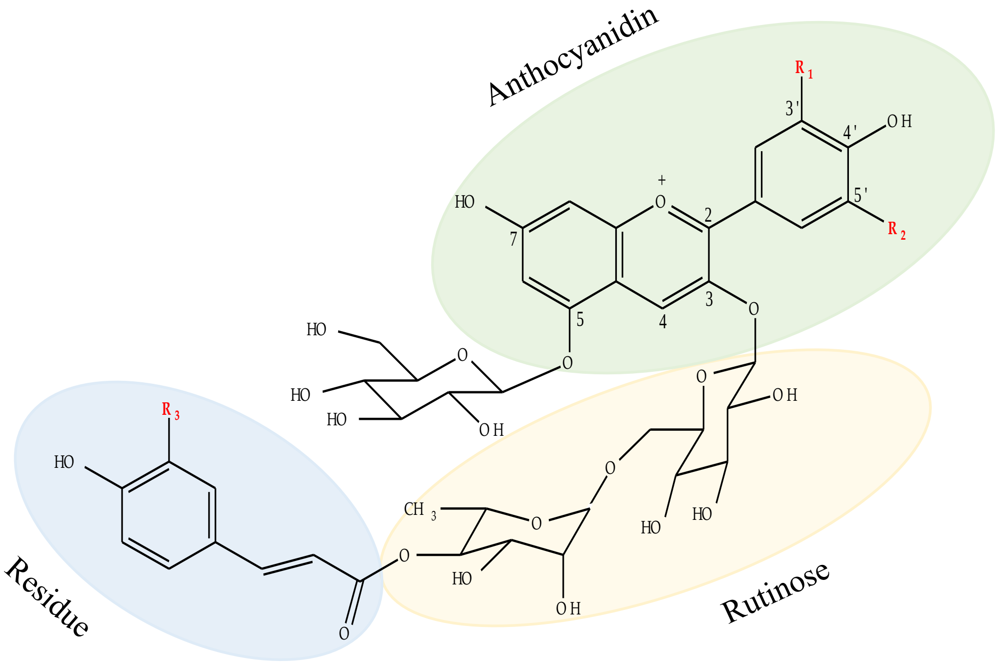

R_1	R_2	**Anthocyanidin**
H	H	Pelargonidin
OCH_3	H	Peonidin
OCH_3	OH	Petunidin
OCH_3	OCH_3	Malvdin

R_3	**Residue**
H	p-Coumaroyl
OH	Caffeoyl
OCH_3	Feruloyl
