# Supplementary material for: Biochemical Characterization and Effects of Cooking Methods on Main Phytochemicals of Red and Purple Potato Tubers, a Natural Functional Food
Source: Foods. 2022 Jan 28;11(3):384. doi: 10.3390/foods11030384 (PMC8834363; doi:10.3390/foods11030384)
Supplement: Supplementary file 1 [file foods-11-00384-s001.zip › Supplementary tables_R1.pdf]

Table S1. Tuber characteristics of the potato genotype tested in the experimental field. For each genotype, the name, origin, tuber skin color (TSC), tuber flesh color (TFC), tuber shape (TS), eye and stolon characteristics are reported.

**Genotype**	**TSC**	**TFC**	**TS**
Musica	Yellow	Yellow	Oblong
Scano di Montiferro	Dark violet	Violet	Oblong irregular
Violet Queen	Dark violet	Violet	Oblong
Magenta Love	Red	Pink	Oblong

Table S2. Anthocyanin profile in raw and cooked potatoes expressed as mg kg^{-1} of DM. Values are means ± SD (n = 3). Statistical analyses were made by comparing anthocyanin content across varieties. Means denoted by the same letter did not differ significantly at $P \leq 0.05$ according to Tukey's multiple range test.

**Variety**		Petunidin 3-O rutinoside	Petunidin 3-O-caffeoyl-rutinoside-5-O-glucoside	Petunidin 3-O-p-coumaroyl rutinoside 5-O-glucoside	Petunidin 3-O-feruloyl rutinoside 5-O-glucoside	Malvidin 3-O-p-coumaroyl-rutinoside-5-O-glucoside	Malvidin 3-O-p-feruloyl-rutinoside-5-O-glucoside
Violet Queen	Fresh non-peeled	19.4 ± 2.0 (b)	27.4 ± 1.3 (c)	360.9 ± 13.0 (b)	17.0 ± 0.8 (c)	98.7 ± 3.2 (b)	12.8 ± 0.9 (a)
	Fresh peeled	17.6 ± 1.0 (b)	60.5 ± 2.9 (c)	912.5 ± 33.9 (b)	31.7 ± 0.4 (c)	177.0 ± 3.8 (b)	22.3 ± 1.6 (b)
	Boiled peeled	28.2 ± 1.1 (b)	183.9 ± 10.7 (b)	3598.7 ± 67.5 (c)	75.0 ± 3.8 (c)	323.8 ± 13.5 (b)	27.5 ± 1.3 (b)
	Microwaved non-peeled cut	47.8 ± 2.3 (b)	216.2 ± 11.9 (b)	2752.7 ± 46.9 (b)	43.6 ± 2.0 (b)	283.0 ± 11.8 (b)	15.9 ± 1.0 (a)
	Baked non-peeled cut	19.0 ± 1.5 (b)	166.6 ± 9.8 (b)	625.4 ± 21.9 (c)	20.6 ± 1.0 (c)	98.4 ± 4.6 (b)	10.5 ± 0.5 (b)
	Fried	14.0 ± 0.9 (b)	75.0 ± 3.0 (b)	528.7 ± 14.7 (c)	15.2 ± 0.9 (c)	72.2 ± 3.6 (b)	8.2 ± 0.8 (b)
Blue star	Fresh non-peeled	< 0.5 (a)	11.3 ± 0.8 (a)	24.8 ± 1.3 (a)	11.4 ± 0.7 (b)	15.5 ± 0.7 (a)	11.5 ± 1.0 (a)
	Fresh peeled	< 0.5 (a)	15.4 ± 0.8 (a)	32.9 ± 1.8 (a)	8.9 ± 0.3 (b)	11.5 ± 0.9 (a)	8.9 ± 0.6 (a)

	Boiled peeled	< 0.5 (a)	10.8 ± 0.6 (a)	23.4 ± 1.0 (a)	10.6 ± 0.4 (b)	11.9 ± 0.6 (a)	9.1 ± 0.9 (a)
	Microwaved non-peeled cut	< 0.5 (a)	51.9 ± 3.0 (a)	308.4 ± 13.0 (a)	70.1 ± 2.1 (c)	47.2 ± 2.1 (a)	28.5 ± 1.1 (b)
	Baked non-peeled cut	< 0.5 (a)	25.6 ± 1.1 (a)	71.7 ± 3.0 (a)	13.9 ± 0.5 (b)	12.2 ± 0.8 (a)	8.0 ± 0.3 (a)
	Fried	< 0.5 (a)	12.9 ± 0.8 (a)	41.7 ± 2.1 (a)	10.3 ± 0.7 (b)	10.3 ± 0.5 (a)	6.5 ± 0.2 (a)
Scano di Montiferro	Fresh non-peeled	31.3 ± 1.8 (c)	16.2 ± 1.0 (b)	13.3 ± 1.0 a	- (a)	350.1 ± 15.5 (c)	29.5 ± 1.9 (b)
	Fresh peeled	36.7 ± 2.0 (c)	21.9 ± 1.1 (b)	42.3 ± 1.4 a	- (a)	443.1 ± 17.3 (c)	34.8 ± 1.9 (c)
	Boiled peeled	45.5 ± 2.8 (c)	16.8 ± 1.0 (a)	172.2 ± 6.4 b	- (a)	690.1 ± 22.9 (c)	66.2 ± 3.6 (c)
	Microwaved non-peeled cut	104.4 ± 4.9 (c)	53.8 ± 3.9 (a)	284.2 ± 11.7 a	- (a)	892.5 ± 17.9 (c)	103.7 ± 2.8 (c)
	Baked non-peeled cut	42.5 ± 1.9 (c)	30.7 ± 1.9 (a)	125.1 ± 7.0 b	- (a)	344.8 ± 18.9 (c)	38.3 ± 2.0 (c)
	Fried	27.2 ± 0.9 (c)	12.9 ± 0.8 (a)	77.1 ± 4.9 b	- (a)	217.7 ± 11.6 ©	17.9 ± 1.0 (c)

Table S3. Anthocyanin profile in raw and cooked potatoes expressed as mg kg^{-1} of DM (mean ± SD; n=3).

**Cultivar**		Petunidin 3-O-rutinoside-5-glucoside	Pelargonidin 3-O-rutinoside	Pelargonidin 3-O-p-caffeoyl-rutinoside-5-O-glucoside	Pelargonidin 3-O-cis-p-coumaroyl-rutinoside-5-O-glucoside	Pelargonidin 3-O-p-coumaroyl-rutinoside-5-O-glucoside	Peonidin 3-O-p-coumaroyl-rutinoside-5-O-glucoside	Pelargonidin 3-O-p-feruloyl-rutinoside-5-O-glucoside	Peonidin 3-O-p-feruloyl-rutinoside-5-O-glucoside
Magenta Love	Fresh non-peeled	< 0.5	< 0.5	23.8 ± 1.1	26.7 ± 2.4	129.1 ± 9.0	30.0 ± 2.0	25.3 ± 1.0	20.6 ± 1.0
	Fresh peeled	54.0 ± 4.1	19.4 ± 1.9	39.0 ± 2.5	82.3 ± 6.5	573.6 ± 21.8	90.2 ± 6.8	42.6 ± 2.9	16.2 ± 0.6
	Boiled peeled	103.9 ± 5.9	31.4 ± 2.8	90.9 ± 7.6	125.3 ± 7.9	1255.0 ± 84.5	158.2 ± 10.0	92.0 ± 7.0	28.8 ± 1.0
	Microwaved non-peeled cut	217.5 ± 9.1	<0.5	151.0 ± 6.9	378.8 ± 12.7	2433.2 ± 71.1	449.6 ± 21.8	118.8 ± 7.3	40.3 ± 2.5
	Baked non-peeled cut	44.2 ± 3.6	13.9 ± 1.2	27.0 ± 2.8	107.6 ± 8.5	353.1 ± 22.6	114.5 ± 5.1	33.2 ± 1.9	15.7 ± 0.8
	Fried	34.6 ± 2.0	8.9 ± 1.1	26.5 ± 3.1	63.0 ± 5.3	410.2 ± 18.9	68.8 ± 4.8	28.3 ± 1.8	9.1 ± 0.5

Table S4. Content of vitamin C in raw and cooked potatoes expressed as mg kg^{-1} of DM (mean ± SD; n=3). Statistical analyses were made by comparing vitamin C content across varieties. Means denoted by the same letter did not differ significantly at $P \leq 0.05$ according to Tukey's multiple range test.

Variety	Fresh non-peeled	Fresh peeled	Boiled peeled	Microwaved non-peeled cut	Baked non-peeled cut	Fried
Musica	597.8 ± 14.1 (a)	524.7 ± 22.7 (a)	345.1 ± 22.3 (a)	311.1 ± 9.9 (a)	299.0 ± 8.2	82.7 ± 2.4
Magenta Love	382.9 ± 21.6 (b)	387.9 ± 8.0 (b)	291.9 ± 4.1 (b)	233.1 ± 4.7 (b)	167.1 ± 9.2	91.2 ± 3.5
Violet Queen	304.3 ± 9.9 (c)	325.5 ± 8.5 (c)	258.8 ± 7.6 (c)	196.3 ± 5.5 (c)	101.9 ± 3.4	36.6 ± 3.7
Blue star	268.3 ± 26.7 (d)	280.7 ± 9.9 (d)	219.3 ± 15.6 (d)	158.3 ± 10.2 (d)	84.8 ± 4.0	32.4 ± 2.5
Scano di Montiferro	307.0 ± 12.6 (c)	316.9 ± 12.6 (c)	204.5 ± 5.4 (d)	135.8 ± 7.2	100.7 ± 2.0	33.1 ± 2.7

Table S5. Content of α-chaconine in raw and cooked potatoes expressed as mg kg^{-1} of DM (mean ± SD; n=3). Statistical analyses were made by comparing α-chaconine content across different varieties. Means denoted by the same letter did not differ significantly at P ≤ 0.05 according to Tukey's multiple range test.

Variety	Fresh non-peeled	Fresh peeled	Boiled peeled	Microwaved non-peeled cut	Baked non-peeled cut	Fried
Musica	141.3 ± 5.5 (a)	9.2 ± 0.6 (a)	8.6 ± 0.5 (a)	99.3 ± 2.8 (a)	62.6 ± 2.4 (a)	6.6 ± 0.5 (a)
Magenta Love	105.2 ± 5.2 (b)	10.6 ± 0.5 (a)	7.6 ± 0.6 (b)	84.7 ± 3.3 (b)	70.6 ± 3.7 (b)	8.3 ± 0.6 (a)
Violet Queen	151.6 ± 3.4 (c)	41.4 ± 2.5 (b)	28.4 ± 1.9 (c)	65.4 ± 4.9 (c)	55.0 ± 4.8 (c)	12.1 ± 1.6 (b)
Blue star	181.0 ± 3.9 (d)	33.1 ± 2.4 (c)	24.7 ± 0.9 (d)	81.1 ± 3.1 (d)	69.1 ± 2.9 (ab)	15.6 ± 0.6 (c)
Scano di Montiferro	166.3 ± 3.4 (e)	38.2 ± 1.7 (d)	42.6 ± 1.8 (d)	80.8 ± 2.1 (d)	74.5 ± 3.9 (b)	12.3 ± 1.5 (b)

Table S6. Content of α-solanine in raw and cooked potatoes expressed as mg kg^{-1} of DM (mean ± SD; n=3). Statistical analyses were made by comparing of α-solanine content across varieties. Means denoted by the same letter did not differ significantly at P ≤ 0.05 according to Tukey's multiple range test.

Cultivar	Fresh non-peeled	Fresh peeled	Boiled peeled	Microwaved non-peeled cut	Baked non-peeled cut	Fried
Musica	98.3 ± 2.0 (a)	7.6 ± 0.4 (a)	7.1 ± 0.2 (a)	50.8 ± 2.0 (a)	53.0 ± 2.7 (a)	6.1 ± 0.4 (a)
Magenta Love	66.8 ± 3.0 (b)	12.9 ± 0.6 (b)	7.5 ± 0.6 (a)	30.0 ± 1.9 (b)	34.1 ± 1.2 (b)	3.8 ± 0.4 (b)
Violet Queen	69.9 ± 3.1 (b)	27.6 ± 2.7 (c)	18.8 ± 1.4 (b)	23.4 ± 2.0 (c)	25.1 ± 1.1 (c)	5.7 ± 0.4 (a)
Blue star	92.9 ± 2.5 (c)	29.5 ± 0.8 (c)	22.0 ± 1.6 (c)	34.6 ± 0.7 (b)	30.1 ± 1.5 (b)	2.7 ± 0.4 (c)
Scano di Montiferro	51.1 ± 3.7 (d)	19.2 ± 1.5 (d)	16.5 ± 1.1 (d)	31.0 ± 1.4 (d)	33.3 ± 1.5 (d)	6.9 ± 0.6 (a)
